# SKI2162, an inhibitor of the TGF-β type I receptor (ALK5), inhibits radiation-induced fibrosis in mice

**DOI:** 10.18632/oncotarget.2878

**Published:** 2015-02-17

**Authors:** Jin-hong Park, Seung-Hee Ryu, Eun Kyung Choi, Seung Do Ahn, Euisun Park, Kyung-Chul Choi, Sang-wook Lee

**Affiliations:** ^1^ Department of Radiation Oncology, University of Ulsan College of Medicine, Asan Medical Center, Seoul, Korea; ^2^ Department of Biomedical Sciences, University of Ulsan College of Medicine, Seoul, Korea; ^3^ Life Science Research Center, SK Chemicals, Seongnam-si, Korea

**Keywords:** Radiation, Fibrosis, TGF-β1, ALK5, SKI2162

## Abstract

Here we demonstrated that SKI2162, a small-molecule inhibitor of the TGF-β type I receptor (ALK5), prevented radiation-induced fibrosis (RIF) in mice. SKI2162 inhibited phosphorylation of Smad and induction of RIF-related genes *in vitro*. In RIF a mouse model, SKI2162 reduced late skin reactions and leg-contracture without jeopardizing the acute skin reaction. Irradiation of mouse tissue increased COL1A2 mRNA levels, and topical administration of SKI2162 significantly inhibited this effect. Thus, these findings support that SKI2162 has potential value as novel RIF-protective agent, and could be candidate for clinical trials.

## INTRODUCTION

Radiation therapy (RT) is among the main cancer treatment modalities together with surgery and chemotherapy, and about half of all newly diagnosed patients will receive RT during the course of their disease [1]. RT is associated with various late complications caused by fibrosis and vascular damage, which are regarded as the main pathological processes of the late RT response [2–4]. Radiation-induced fibrosis (RIF) in skin and soft tissue, which is characterized by excessive accumulation of extracellular matrix and the proliferation of fibroblasts, is one of the most common late complications of RT [2, 3]. Although RIF was traditionally regarded as an irreversible process that leads to dead fibrous tissue, RIF is now recognized as a dynamic process related to the remodeling of scar tissue by continuously reactivated myofibroblasts [5].

Transforming growth factor beta (TGF-β) is the main signaling molecule in fibrosis. Radiation can activate the TGF-β signaling pathway, and continuous expression of TGF-β has been observed in the early and late phases of RIF [6, 7]. This signaling pathway involves TGF-β binding to the TGF-β type II receptor, which recruits the TGF-β type I receptor, also known as ALK5 (activin receptor-like kinase-5), resulting in the assembly of a heterodimeric receptor complex [8]. This receptor complex phosphorylates the Smad proteins; the activated Smad complex then translocates to the nucleus where the Smad proteins bind to their DNA binding site to initiate gene expression [8, 9]. Therefore, inhibition of the TGF-β signaling pathway could be a very effective strategy for controlling fibrosis. There have been numerous attempts to develop anti-fibrotic agents targeting the TGF-β signaling pathway, including TGF-β-neutralizing antibodies, antisense oligonucleotides against TGF-β, and TGF-β receptor antagonists [10].

Recently, SKI2162, a novel small-molecule inhibitor of ALK5, was synthesized. SKI2162, which competitively inhibits the ATP binding site of ALK5 and is highly selective for ALK5, is being developed for the treatment of RIF. SKI2162 was also recently reported to block TGF-β1-induced phosphorylation and nuclear translocation of Smad2 and Smad3 [11]. In the this study, we evaluated the inhibitory effect of SKI2162 against the TGF-β signaling pathway *in vitro* as well as *in vivo* using our previously established RIF mouse model [6].

## RESULTS

### Inhibitory and selectivity effects of SKI2162 on ALK5

**Figure 1:**
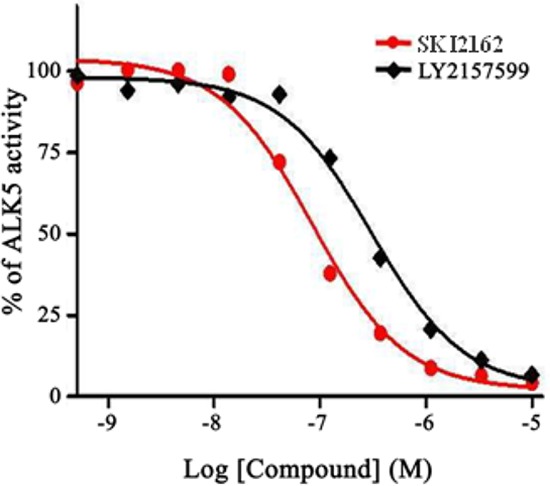
Concentration-dependent effects of SKI2162 on ALK5 inhibition The inhibition of ALK5 activity by SKI2162 and LY2157299 was tested using purified recombinant ALK5. Different concentrations of each test compound were used and percent-activity values were calculated to derive the corresponding IC_50_ values. The calculated IC_50_ values for SKI2162 and LY2157299 were 0.094 μM and 0.327 μM, respectively.

The result of assays for inhibitory and selectivity effects of SKI2162 on ALK5 are shown in Figure 1 and Supplementary Table 1. The IC_50_ values for SKI2162 and LY2157299 were 0.094 μM and 0.327 μM, respectively, demonstrating that SKI2162 was approximately 3-fold more potent than LY2157299 in inhibiting ALK5 activity. Selectivity assays of SKI2162 and LY2157299 indicated that SKI2162 was 21-fold more selective for ALK5 inhibition than p38 MAPK inhibition, whereas LY2157299 did not discriminate between ALK5 and p38 MAPK. Both SKI2162 and LY2157299 showed 73-fold and 40-fold higher selectivity for ALK5 than ALK1, respectively.

### Activation of Smad2 and Smad3 by TGF-β1 is directly inhibited by SKI2162 in keratinocyte and fibroblast cells

**Figure 2:**
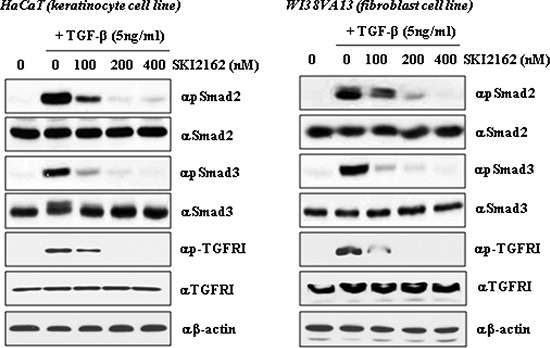
The effect of SKI2162 on TGF-β1-induced activation of Smad2 and Smad3 in HaCaT (keratinocyte cell) and WI38VA13 (fibroblast cell) cells The induction of p-Smasd2, p-Smad3 and p-TGF-β receptor I expression by TGF-β1 was repressed by SKI2162 treatment in HaCaT cells and WI38VA13 cells.

The inhibitory effects of SKI2162 on phosphorylation of Smad proteins were evaluated in HaCaT and WI38VA13 cells by western blotting. Cells were pretreated for 1 h with increasing concentration of SKI2162 (0, 100, 200, and 400 nM), after which TGF-β1 (5 ng/ml) was added and the cells were incubated for 1 h. A representative western blot of pSmad2, Smad2, pSmad3, and Smad3 are shown in Figure 2. TGF-β1 increased the levels of phosphorylated Smad2 and Smad3 in both cells and pre-incubation with SKI2162 diminished their phosphorylation in response to TGF-β1 in a dose-dependent manner. Phosphorylation of both Smad2 and Smad3 was almost completely abolished at 400 nM SKI2162.

### SKI2162 down-regulates RIF markers in fibroblast cell

**Figure 3:**
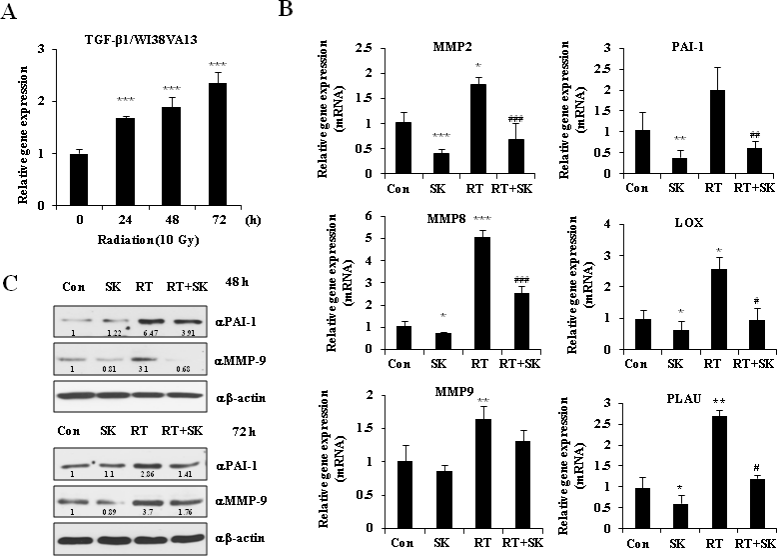
SKI2162 (SK), an inhibitor of ALK5, suppressed radiation-induced fibrosis (RIF)-related genes **(A)** The mRNA expression of TGF-β1 induced by radiation in the fibroblast cell lines (***p < 0.001 *vs*. 0 hour). **(B)** Reduced gene expression level of RIF-related markers by SKI2162 (*p < 0.05, **p < 0.01, and ***p < 0.001 *vs*. control, ^#^*p* < 0.05, ^##^*p* < 0.01, ^###^*p* < 0.001 *vs*. RT). **(C)** Reduced induction of the RIF-related proteins (PAI-1 and MMP9) by SKI2162.

To determine whether SKI2162 regulates radiation-induced TGF-β1 activation and the TGF- β1-mediated fibrosis response, TGF-β1 mRNA expression was evaluated in human WI38VA13 fibroblast cells by real-time PCR following irradiation (10 Gy). As shown in Figure 3A, the level of TGF-β1 mRNA significantly increased after irradiation in a time-dependent manner. Due to the important role of TGF-β1 in the regulation of fibrogenesis, the effect of SKI2162 on the radiation-induced transcription of TGF-β1-regulared genes, particularly fibrosis genes, was evaluated using real-time PCR. As shown in Figure 3B, SKI2162 treatment significantly reduced the radiation-induced transcriptional expression of MMP2, MMP8, PAI-1, LOX and PLAU. Similar results were also observed in TGF-β1-treated HaCaT cells (Supplementary Figure 1). PAI-1 and MMP9 were also increased by radiation at the protein level(Supplementary Figure 2), and SKI2162 inhibited this effect (Figure 3C).

### SKI2162 inhibits RIF in mouse model

**Figure 4:**
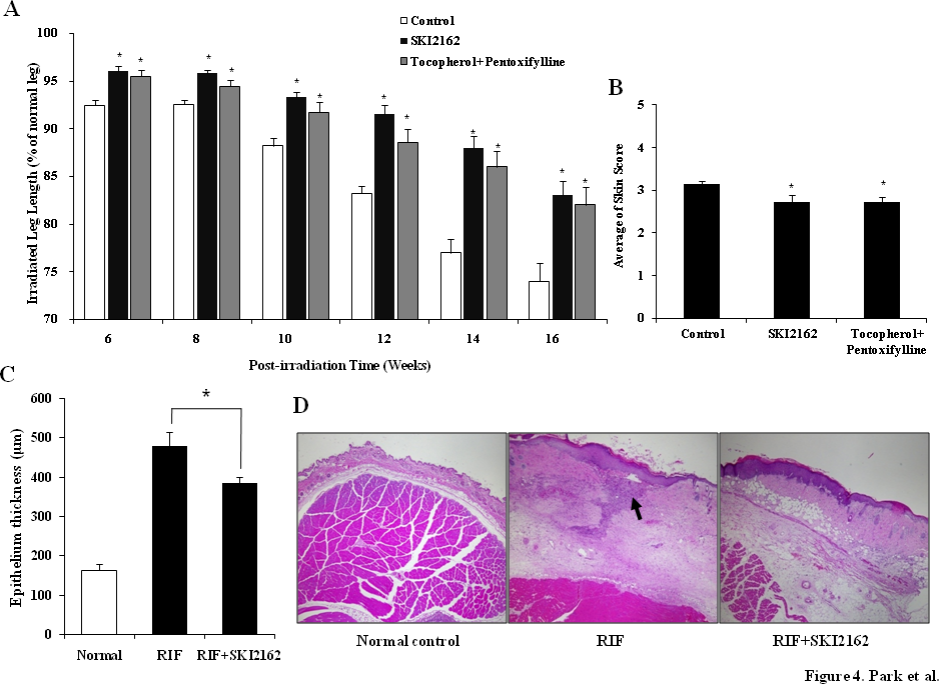
SKI2162 inhibited radiation-induced fibrosis (RIF) in a mouse model **(A)** Leg-contracture assay results by SKI2162 treatment in the RIF mouse model (*p < 0.05 *vs*. control). **(B)** Late skin reaction score in the RIF mouse model (*p < 0.05 *vs*. control). **(C)** Epithelial thickness by treatment group (control and SKI2162 group only). **(D)** H&E staining of epithelial tissues. Radiation induced dramatic changes especially in both epidermis and dermis compared to normal skin and the treatment of SKI2162 ameliorated radiation induced skin damages.

To investigate the anti-fibrotic effect of SKI2162 *in vivo*, a RIF mouse model was used. In a leg-contraction assay (Figure 4A), the mean length of the irradiated leg was significantly greater in the SKI2162 and tocopherol-pentoxifylline (PTX) treated groups than in the control group between 6 and 16 weeks post-irradiation. Early skin reactions were measured during the third and fourth weeks post-irradiation. The early skin reaction grades were almost identical among the three groups (data not shown). Late skin reaction scores measured at 16 weeks post-irradiation were 3.14 ± 0.08 in the control group, 2.74 ± 0.11 in the tocopherol-PTX group, and 2.73 ± 0.16 in the SKI2162 group (Figure 4B). Both treated groups showed significantly lower levels of skin reaction than the control group (*p* = 0.034 for the tocopherol-PTX group and *p* = 0.006 for the SKI2162 group) (Supplementary Table 2). SKI2162 was well tolerated and neither treatment group showed significant differences in body weight compared with the RIF group (saline) at any post-irradiation time point (Data not shown). To investigate the effect of SKI2162 in the skin and soft tissue of the irradiated leg, the epithelial thickness from the surface of the epidermis to the base of the dermis was measured. As shown in Figure 4C, the thickness of the epithelium was greater in the control group (saline) than in the SKI2162 group (479.06 ± 35.51 μm vs. 385.27 ± 15.55 μm, *p* = 0.035 by a one-sided test), suggesting that SKI2162 blocked collagen accumulation and fibrotic changes. We also checked the histology of untreated normal skin and the epithelium thickness was 163.11 ± 16.46 μm. As shown in H&E images (Figure 4D), radiation induced dramatic changes especially in both epidermis and dermis compared to normal skin and the treatment of SKI2162 ameliorated radiation induced skin damages such as necrosis and collagen accumulation.

**Figure 5:**
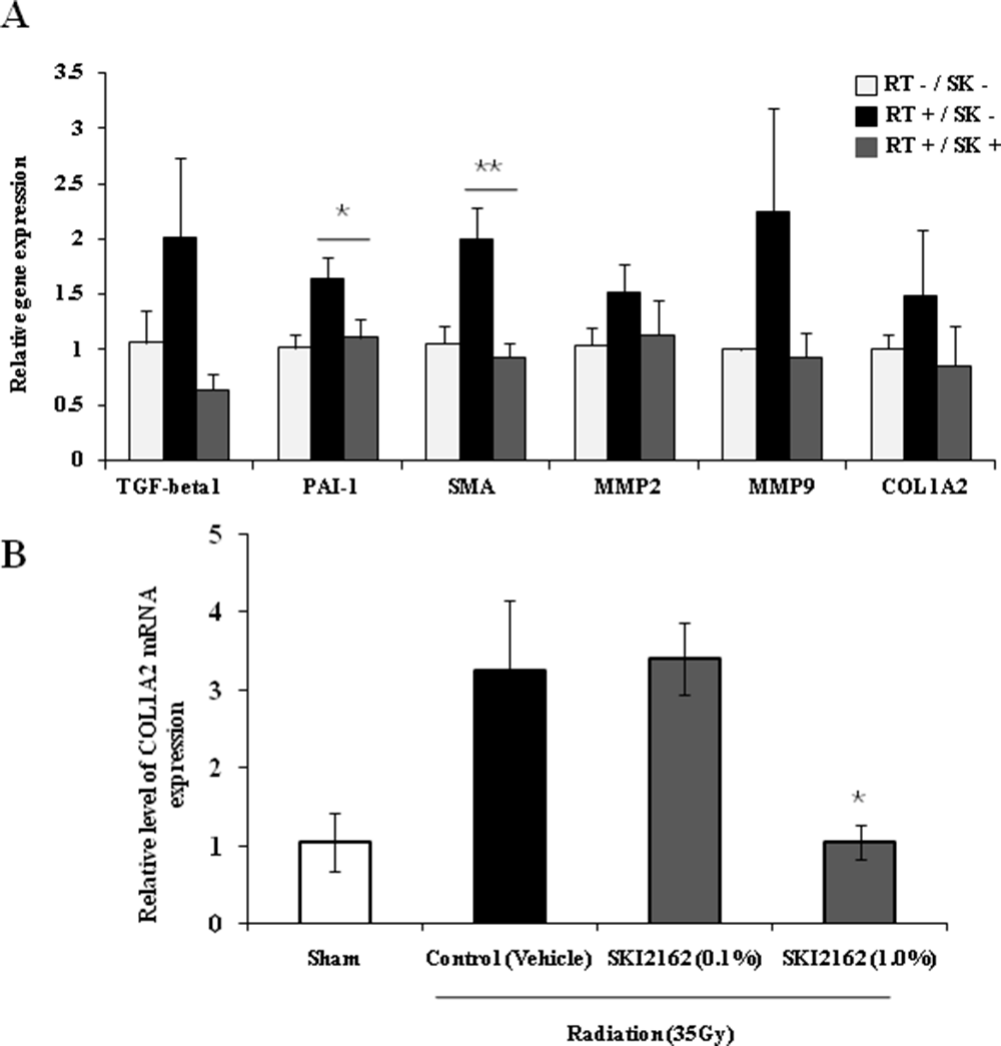
SKI2162 (SK) suppressed fibrosis-induced target genes in the radiation-induced fibrosis (RIF) mouse model **(A)** Real-time PCR showed that RIF-related markers were inhibited by SKI2162 (*p < 0.05, **p < 0.01 *vs*. RT+/SK−). **(B)** Topical administration of SKI2162 (1.0%) significantly repressed the induction of COL1A2 mRNA in a RIF mouse model (*p < 0.05 *vs*. control).

To demonstrate the inhibitory role of SKI2162 in a RIF mouse model, the expression of fibrosis-related target genes was examined by real-time PCR using mRNA from the legs of irradiated mice with or without SKI2162 at 16 weeks post-irradiation. A significant reduction in the radiation-induced transcription of PAI-1 (*p* = 0.042) and SMA (*p* = 0.0028) were observed following SKI2162 treatment (Figure 5A). Next, to assess the efficacy of topical application of SKI2162, COL1A2 mRNA expression was evaluated in a RIF mouse model (Figure 5B). A single radiation dose of 35 Gy was delivered to the hind limb of each mouse to induce skin fibrosis and SKI2162 (0.1% or 1.0%) or vehicle was topically applied for 14 days post-radiation. COL1A2 mRNA levels increased in vehicle-treated mice compared to sham-treated mice suggesting that radiation induces up-regulation of collagen synthesis 14 day post-irradiation. Furthermore, COL1A2 mRNA was significantly decreased in mice treated with 1.0% SKI2162 compared to sham-treated controls (*p* = 0.016).

## DISCUSSION

The present study showed that the activation of the TGF-β signaling pathway by radiation, which has been regarded as a major mechanism in the pathogenesis of RIF, was effectively controlled by SK2162 (an ALK5 inhibitor), leading to the amelioration of RIF in a mouse model. Among the various signaling molecules involved in fibrosis, TGF-β1 has been described as the master switch for the fibrotic mechanism [3–5]. TGF-β1 is a multi-functional cytokine associated with cell proliferation, cell differentiation, cell migration, production of extracellular material, and immune reactions; in particular, it plays a central role in fibroblast proliferation and the production of collagen fibers [17]. After TGF-β binds its receptor, the activated TGF-β receptor phosphorylates the Smad proteins, and the activated Smad complex translocates to the nucleus where the Smad proteins bind to their DNA binding site to initiate gene expression [5]. Notably, disruption of the Smad3 gene in knockout mice provides significant protection from radiation-induced skin injury [18].

It is well known that radiation can directly activate the TGF-β signaling pathway [6, 7]. Even very low-dose radiation (0.1 Gy) can induce TGF-β activation and radiation-induced stromal changes, and a dose-response relationship up to 5 Gy has been observed [19]. Also, induction of TGF-β signaling is initiated quickly after irradiation. Martin et al. showed that induction of TGF-β is detectable in skin tissue 6 h after single radiation doses ranging from 16 to 64 Gy [20]. Moreover, TGF-β1 mRNA was overexpressed during the early erythematous phase and activation of TGF-β1 continued from 6 to 12 months after irradiation in a porcine model of RIF. Similar alterations have been detected as long as 27 years after irradiation in radiation-induced human skin fibrosis [21]. These observations indicate that RIF is not a stationary environment resulting from temporal alteration of the normal repair mechanism, but rather a dynamic process with vicious cycles of fibrosis and inflammation associated with TGF-β signaling. Given that the TGF-β signaling pathway is a central mechanism in fibrotic changes, targeting this pathway, and thereby blocking the vicious cycle of TGF-β1 signaling, is a reasonable strategy for protecting against RIF.

In the present study, the TGF-β1 receptor was used as the target for RIF protection. As shown in our study, SKI2162, a newly developed novel small-molecule inhibitor of the TGF-β1 receptor (ALK5) is a more potent and selective inhibitor of the TGF-β1 receptor than LY2157299, a potent ALK5 inhibitor that has been used in clinical trials [12]. And our study clearly indicated that SKI2162 blocked the TGF-β signaling pathway and fibrosis-related gene expressions. Western blot analyses demonstrated that phosphorylated Smad2 and Smad3, which are the activated forms of these proteins, were decreased in a concentration-dependent manner by SKI2162 treatment. Also, the induction of fibrosis-related genes was significantly inhibited by SKI2162 treatment of fibroblast cells. Additionally, topically applied SKI2162 inhibited the induction of COL1A2 mRNA in a RIF mouse model. Remarkably, SKI2162 could inhibit PAI-1 expression by blocking TGF-β signaling in irradiated cells and tissues. In irradiated tissues, cells generate reactive oxygen species (ROS) and elevated ROS promotes fibrogenesis via activation of TGF-β which stimulates collagen as well as PAI-1. PAI-1 gene expression is tightly regulated by a variety of cytokines such as TGF-β, interleukin-1β, and TNF-α, and the PAI-1 promoter contains important regulator elements including Smad2, Smad3, CREB-binding protein (CBP) and specificity protein 1 (sp1) [22–24]. PAI-1 is considered to be an important inhibitor of fibrinolysis and the direct accumulation of extracelluar matrix, which suggests it plays a pivotal role in the development of tissue fibrosis [25].

Also, predisposing genetic factors might play major role in the diversity of development of RIF. Several reports showed fibrosis-related basal gene expression level could be related with a RIF. Forrester HB et al. compared the basal expression profiles between cell lines of cancer patients with and without severe fibrosis using Exon arrays. They identified candidate genes related with RIF prediction and these genes are associated with TGF-β and retinoic acid which have a role in the fibrogenic process [28]. Andreassen CN et al. conducted study for validation of a previously established predictive test for the risk of RIF based on the gene expression pattern. They classified 160 patients with head and neck cancer who received RT into sensitive or resistant expression profile. The cumulative risk of fibrosis was 34% at 9 years for the patients with sensitive profile opposed to 0% for resistant profile [29].

The *in vivo* efficacy of SKI2162 was confirmed in a RIF mouse model. The leg-contracture assay showed a significant protective effect in the SKI2162 treatment group in the 16 weeks after irradiation. This finding suggested that TGF-β1-related dynamic processes of fibrosis might continue in the control group up to 4 months post-irradiation and SKI2162 showed a continuous protective effect during the same period. These results are in agreement with our previous finding that increased TGF-β mRNA could be detected in irradiated tissue up to 3 months post-irradiation [6]. Also, although SKI2162 had no significant effects on acute skin reactions, the SKI2162 treatment group showed a significantly lower level of late skin reactions than the control group. This suggests that inhibition of the TGF-β signaling pathway by SKI2162 has a more potent effect on the late phase of fibrosis than on the initial phase. We also tested the possibility of topical application of SKI2162 in radiation-induced fibrosis mouse model. However it takes several months to develop skin fibrosis, we simply modified irradiation schedule to mimic similar skin condition. Single radiation dose of 35 Gy was delivered to induce the increased gene expression of COL1A2 and topical treatment of 1% SKI2162 for 14 days completely inhibited COL1A2 induction compared to sham-treated control. Even though RIF is considered as long-term process, this experiment could provide meaningful information.

Vitamin E (Vit E) plus PTX is one of the most widely studied anti-fibrotic treatment regimens for superficial fibrotic lesions [13]. In one randomized, placebo-controlled clinical trial, Vit E plus PTX was reported to significantly reduce RIF in patients who had received RT for breast cancer [26]. In the current study, we confirmed the antifibrotic effect of Vit E plus PTX in the RIF mouse model, showing that radiation-induced leg contracture was significantly reduced in the Vit E plus PTX-treated group from 6 to 16 weeks post-irradiation. The Vit E plus PTX-treated group also showed more favorable late skin reactions than the control group (Supplementary Figure 4). Although a comparison showed a trend toward greater protection by SKI2162, the difference between the protective effect of SKI2162 and Vit E plus PTX was not significantly different. Several molecular mechanisms for the effect of Vit E and PTX were suggested, but exact mode of action of these agents is unknown. Recently, Hamama S et al. investigated the activation of the TGF-β/Smad and Rho/ROCK pathways using primary smooth muscle cells isolated from intestinal samples from humans with radiation enteropathy which were incubated with Vit E and PTX. Hamama S et al. showed that PTX and Vit E suppress fibrogenic action by inhibition of TGF-β transcription, not by Rho/ROCK pathway [27].

Although the TGF-β signaling pathway is related to exaggerated fibrotic changes, basically, it normally serves a major healing function in tissue injury. Accordingly, we were initially concerned about the possibility that inhibition of TGF-β signaling by SKI2162 might negatively affect healing during the acute skin reaction. However, because induction of the TGF-β signal starts immediately after radiation (Supplementary Figure 5) [20], we assumed that immediate SKI2162 treatment would be more effective and chose not to delay SKI2162 treatment. Contrary to our worst-case expectations, skin-reaction scoring showed no difference in acute skin reactions between the SKI2162 treated group and the control group. One interpretation of these results is that TGF-β signaling through ALK5 does not entirely account for the normal healing mechanism. TGF-β could act through multiple pathways to regulate healing process, and there is the possibility of cross-talk between the TGF-β signaling pathway and other signaling pathway [30]. Another concern associated with inhibition of the TGF-β pathway for RIF protection is adverse systemic effects. TGF-β is a highly pleiotropic cytokine that contributes to various essential functions including apoptosis control, angiogenesis, wound healing, and immune regulation [31]. Although, we used systemic delivery of SKI2162 (intraperitoneal injection) in the leg-contracture assays to maximize drug effects for validation purpose, there were no definite toxic effects related to drug administration in the RIF mouse model. However, there remains a need for further study about possible toxic effect associated with this agent.

For assessment of anti-fibrotic treatment, it is important to demonstrate this therapeutic effect *in vivo* because the effect of anti-fibrotic treatment at the molecular level may not translate into clinical effect. In the present study, we performed a leg-contracture assay using a Lucite jig, which is an effective, widely accepted method for measuring RIF in mouse models and has an accuracy of 1 ± 0.5 mm [15, 16]. However, results obtained using this method could confounded by inter-observer variation because the measurement of the leg-contracture using a Lucite jig depends on the operator's skill. Although, to minimize error, only one investigator (J. Park) performed this assay with blinded method, a reliable and objective measurement method must be developed for the study of anti-fibrotic therapy.

In conclusion, we assessed the inhibitory effect of SKI2162, a newly developed small-molecule inhibitor of the TGF-β1 type I receptor (ALK5), and assessed its protective effect against RIF using *in vivo* and *in vitro* models. Although many TGF-β receptor-antagonizing drugs have been tested, to the best of our knowledge, the current study is the first report of a small-molecule inhibitor of the TGF-β1 receptor that protects against RIF in a mouse model. SKI2162 could be considered a candidate for future clinical trials of RIF.

## MATERIALS AND METHODS

### ALK5 inhibition and selectivity assay

The IC_50_ for SKI2162 was determined using a radioisotope-based profiling assay and compared to that of LY2157299, a potent, specific ALK5 inhibitor [12]. The kinase activity of ALK5, ALK1, and p38 MAPK were assessed by measuring radiolabelled phosphate (^33^P) incorporation into casein. ALK1 and p38 MAPK are closely related to ALK5, and were used for comparison with ALK5 in a SKI2162 selectivity study. IC_50_ values were calculated from dose–response curves.

### Western blot analysis

Cell culture and reagents are shown in Figures and Supplementary Figures. To assess the effect of SKI2162 on TGF-β1-induced activation of Smad2 and Smad3, HaCaT and WI38VA13 cells were pretreated with increasing concentrations of SKI2162 for 1 h and then TGF-β1 (5 ng/ml) was added for an additional hour. Cell extracts were lysed with lysis buffer and protein expression was detected by conventional western blot analysis. Anti-phospho-Smad2 (Ser465/467), anti-Smad2, anti-phospho-Smad3 (Ser423/425), anti-Smad3, TGF-β Type I receptor and anti-phospho-TGF-β Type I receptor antibodies were used (Cell Signaling Technology, USA). In addition, WI38VA13 cells were pretreated with 200 nM of SKI2162 for 1 h and then irradiated (10 Gy) using a linear accelerator (Varian Medical Systems, USA). Cells were incubated for 48 and 72 h and then analyzed by western blot using plasminogen activator inhibitor 1 (PAI-1) and matrix metalloproteinase (MMP)9 antibodies (Santa Cruz Biotechnology, USA).

### RNA extraction and real-time PCR

The expression of fibrosis-related genes, including MMP2, MMP8, MMP9, PAI-1, lysil oxydase (LOX) and urokinase plasminogen activator gene (PLAU), was measured by real-time PCR following radiation and SKI2162 treatment. WI38VA13 cells were pretreated with or without 200 nM of SKI2162 for 1 h and then irradiated (10 Gy). More detailed method for measurement is shown in Figure 3B.

### Anti-fibrotic effect of SKI2162 in a RIF mouse model

Male BALB/c mice (Central Laboratory Animal, Korea) were used for the RIF mouse model [6]. Under anesthesia, the left hind limb of each mouse received two weekly radiation doses of 22 Gy using a linear accelerator. After irradiation, mice were randomly divided into three groups. Each group was treated with once-daily (5 times/week) intraperitoneal injections of saline (*n* = 20), SKI2162 (*n* = 21; 10 mg/kg), or PTX (*n* = 20; 30 mg/kg, Sigma-Aldrich, USA) plus orally administered tocopherol (DL-alpha-tocopherol; 10 mg/kg; Shinil Pharmaceutical Co., Korea). All treatments were initiated after irradiation and continued for 16 weeks. Tocopherol-PTX is one of the most widely studied anti-fibrotic regimens [13]. The early skin reaction was measured at 3–4-day intervals during 4 weeks after the initial irradiation, and the late skin reaction was evaluated 16 weeks after irradiation. Early and late skin reactions were scored using a grading system (supplementary table 2) described by Dion et al [14]. To assess RIF in mouse model, Lucite jig which was modified from the one described by Stone [15]. The leg-contracture assay was performed as described by Ishii [16], every 2 weeks for 6–16 weeks after irradiation. Briefly, an anesthetized mouse was placed in a Lucite jig, and the length of the extended leg was measured with a ruler inlaid within the base of the jig. The degree of contraction was recorded as the length of the irradiated leg which compared with that of the un-irradiated contralateral leg, expressed as a percentage (Supplementary Figure 3). Mice were sacrificed for histopathologic evaluation and measurement of fibrosis-related gene expression 16 weeks after radiation. More detailed method for measurement is shown in Figure 4A.

### Topical application of SKI2162 and COL1A2 mRNA expression in RIF

The effect of topically applied SKI2162 was also tested in a RIF mice model. In this experiment, one radiation treatment of 35 Gy was administered, and the other conditions were the same as previously described. After irradiation, a total of 18 mice were randomly divided into three groups: a low-dose SKI2162 group (0.1%, *n* = 6), a high dose SKI2162 group (1.0%, *n* = 6), and a vehicle control (*n* = 6). Two mice that received no radiation or treatment were used as a sham control group. Vehicle only (95% propylene glycol, 5% polyethylene glycol) or SKI2162 (0.1% or 1% in vehicle) was topically administered twice daily at 12 h intervals to the site of radiation. On day 14, the skin was excised and total RNA was extracted using the RNeasy Fibrous Tissue Kit (Qiagen, USA). Procedures for cDNA preparation, real-time PCR and analysis were as described above. Primers for amplification are shown in the supplementary Methods and Materials.

### Statistical analysis

All values are presented as means ± standard errors of mean (SEM). Significance was analyzed using the Student's t-test and the Mann-Whitney test. Probability (*p*) values < 0.05 were considered to indicate significant differences.

## SUPPLEMENTARY METHODS AND MATERIALS, FIGURES AND TABLES


