# Supplementary material for: SKI2162, an inhibitor of the TGF-β type I receptor (ALK5), inhibits radiation-induced fibrosis in mice
Source: Oncotarget. 2015 Feb 17;6(6):4171–9. doi: 10.18632/oncotarget.2878 (PMC4414180; doi:10.18632/oncotarget.2878)
Supplement: Supplementary file 1 [file oncotarget-06-4171-s001.pdf]

SUPPLEMENTARY METHODS AND MATERIALS

Cell culture and reagents

A human keratinocyte cell line (HaCaT) and an SV40-transformed human fibroblast cell line (WI38VA13) were cultured in Minimum Essential Media (Gibco/Life Technologies, USA) or in Dulbecco's Modified Eagle's Medium (Gibco/Life Technologies, USA), respectively, with 10% fetal bovine serum (Gibco/Life Technologies, USA), in a humidified 5% CO_2 atmosphere at 37°C. Recombinant human TGF-β1 was purchased from R&D Systems (Minneapolis, USA) and SKI2162 was a kind gift from SK Chemical (Seongnam-si, Korea).

ALK5 inhibition and selectivity assay

For these analyses, GST/histidine tag fused p38 MAPK, ALK1, and ALK5 were expressed in insect cells. Purified proteins were mixed with casein substrate (final concentration, 2 mg/mL) and added to reaction buffer. SKI2162 was prepared with 100% DMSO and added to reaction mixtures. The reaction was initiated by adding ^{33}P-ATP (specific activity 0.01 µCi/µL), and incubated at room temperature for 2 h. After incubation, reactions were spotted onto P81 ion exchange paper and the filters were washed extensively in 0.75% phosphoric acid. The Flash-Plates were then air-dried and counted.

RNA extraction and quantitative real-time PCR

Cells were incubated for 48 h and RNA was isolated with the RNA Easyspin kit according to the instructions of the manufacturer (Intron, Korea). Total RNA was used to prepare cDNA using the PrimeScriptTM reverse transcriptase kit (TaKaRa Bio Inc., Japan), and targets were amplified by real-time PCR using SYBR Green Master mix (Roche, USA). Primers for amplification were as follows: TGF-β1, 5′-AAGTGGACATCAACGGGTTC-3′ (forward) and 5′-TGCGAAGTCAATGTACAGC-3′ (reverse); MMP2, 5′-ACATCAAGGGCATTCAGGAG-3′ (forward) and 5′-GCCTCGTATACCGCATCAAT-3′ (reverse); PAI-1, 5′-CTCTCTCTGCCCTCACCAAC-3′ (forward) and 5′-GTGGAGAGGCTCTTGGTCTG-3′ (reverse); MMP8, 5′-ATTTCCAAGGCCTTTCCTGT-3′ (forward) and 5′-GGGTTTCCTGGGGTTAACAT-3′ (reverse); LOX, 5′-CCAGAGGAGAGTGGCTGAAG-3′ (forward) and 5′-CCAGGTAGCTGGGGTTTACA-3′ (reverse); MMP9, 5′-CATCGTCATCCAGTTTGGTG-3′ (forward) and 5′-TCGAAGATGAAGGGGAAGTG-3′ (reverse); PLAU, 5′-GTCACCACCAAAATGCTGTG-3′ (forward) and 5′-AGGCCATTCTCTTCCTTGGT-3′ (reverse); and GAPDH, 5′-GATGGCATGGACTGTGGTCA-3′ (forward) and 5′-GCAATGCCTCCTGCACCACC-3′ (reverse). All samples were normalized to GAPDH and expressed as the fold induction. Relative expression levels were calculated using the comparative threshold method. All reactions were done in triplicate.

Histopathologic evaluation and fibrosis-related gene expression in RIF tissues

The skin and soft tissue of the irradiated leg was formalin-fixed, paraffin-embedded, cut into 5-µm sections, and stained with hematoxylin-eosin. In each mouse tissue sample, 10 sites were randomly selected for measurement of epithelial thickness, and the mean value was calculated. In addition, immunohistochemistry was conducted to compare the expression levels of p-SMAD2 in the tissues. Briefly, paraffin-embedded slides were submitted to deparaffinization in xylene and hydration with a series of alcohol. Antigen retrieval was conducted using citrate buffer, after then endogenous peroxidase activity was blocked with 3% of hydrogen peroxide for 15 minutes. Slides were incubated with polyclonal anti-phospho-SMAD2 antibody (Cell signaling) overnight. Washed slides were incubated with horseradish peroxidase reagent for one hour, detected by DAB solution in Dako Real EnVision kit (Dako, CA, USA), and then counterstained with hematoxylin. Fibrosis-related gene expression was also compared in skin tissues obtained 16 weeks after radiation. Methods for RNA extraction and real-time PCR were the same as described above. Primers for amplification were as follows: mouse TGF-β1, 5′-GTCCTTGCCCTCTACAACCA-3′ (forward) and 5′-GTTGGACAACTGCTCCACCT-3′ (reverse); mouse PAI-1, 5′-GGAAGAAGACCCGATCAACA-3′ (forward) and 5′-GCCACGAGAATCAAATCCAT-3′ (reverse); mouse α-SMA, 5′-TGTGCTGGACTCTGGAGATG-3′ (forward) and 5′-GAAGGAATAGCCACGCTCAG-3′ (reverse); mouse MMP2, 5′-GAAACCGTGGATGATGCTTT-3′ (forward) and 5′-CCATCAGCGTTCCCATACTT-3′ (reverse); mouse MMP9, 5′-AACACCACCGAGCTATCCAC-3′ (forward) and 5′-AGGAGTCTGGGGTCTGGTTT-3′ (reverse); mouse COL1A2, 5′-AACACCACCGAGCTATCCAG-3′ (forward) and 5′-AGGAGTCTGGGGTCTGGTTT-3′ (reverse). All samples were normalized to GAPDH and expressed as the fold induction. Relative expression levels were calculated using the comparative threshold method.

Topical application of SKI2162 and COL1A2 mRNA expression in RIF

Mouse COL1A2 was amplified using the primers 3′-CAATGGTGGCAGCCAGTTTG-5′ (forward) and 3′-CCAGGTACGCAATGCTGTTCTT-5′ (reverse).

TGF-β1 ELISA assay

TGF-β1 expression was evaluated using Human/Mouse TGF-beta 1 ELISA Ready-SET-Go! kits (eBioscience, USA) according to the manufacturer's instructions. Briefly, The culture media of HaCaT and WI38VA13 cells was concentrated by Centricon (Millipore, USA), and concenrated samples were plated in 96-well coated-plates with antibody. Substrate solution was added to each well, and The plate was incubated in R.T. for 15 min. After stop solution was added, the plate was measured using a SpectraMAX 250 Optima plate reader at 450 nm (Molecular Device Co., Sunnyvale, CA).

SUPPLEMENTARY FIGURES AND TABLES

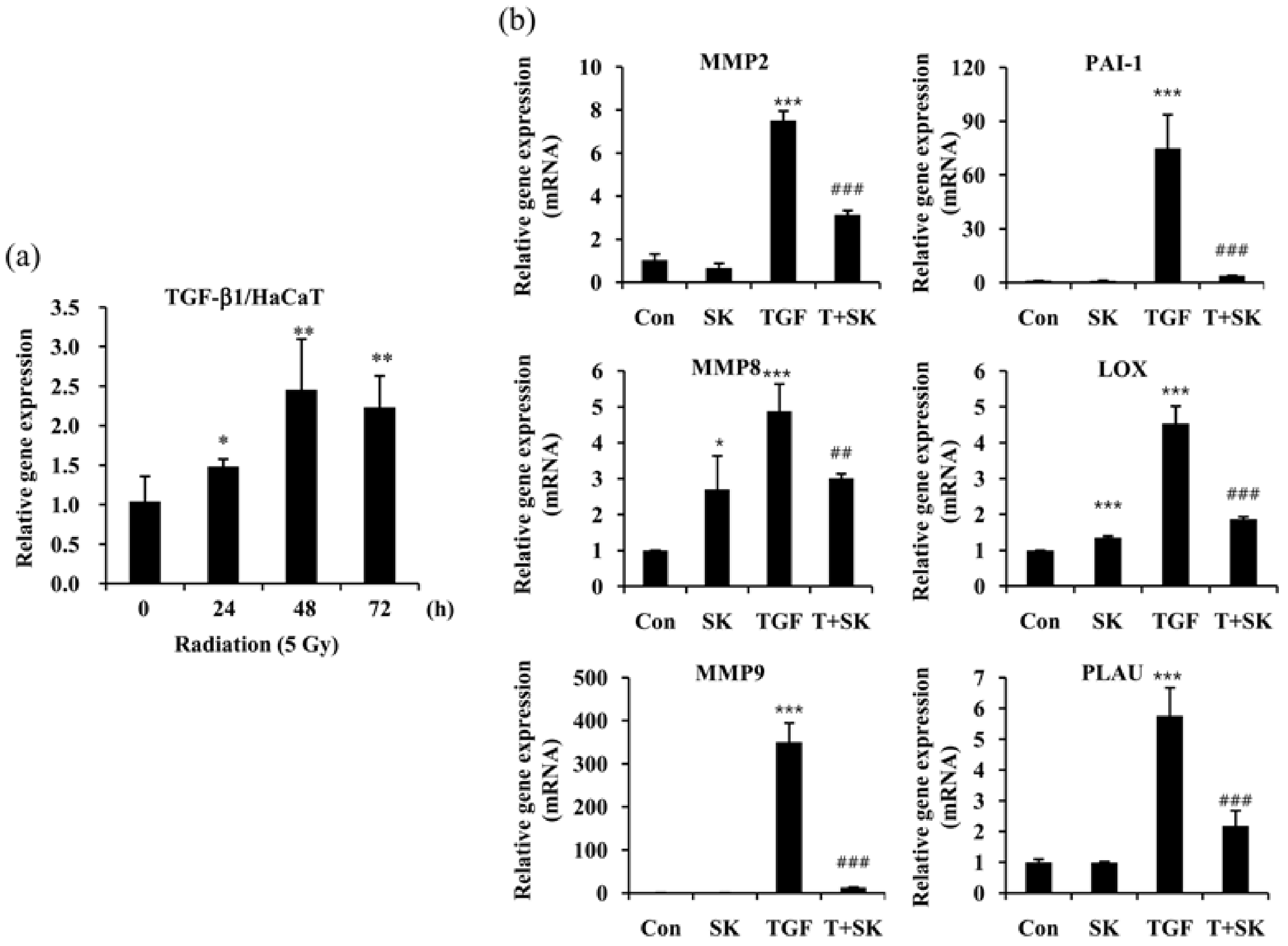

Supplementary Figure 1: SKI2162 suppressed the TGF-β-induced fibrosis related genes in HaCaT cell lines. (a) TGF-β1 is induced by radiation in keratinocyte cells (*$p < 0.05$, **$p < 0.01$). HaCaT cells were treated with radiation (5 Gy) in a time-dependent manner. The mRNA expression of TGF-β1 was analyzed by real-time PCR. **(b)** SKI2162 inhibited TGF-β-induced fibrosis markers. (*$p < 0.05$ and ***$p < 0.001$ *vs.* control; $^{\#\#}p < 0.01$ and $^{\#\#\#}p < 0.001$ *vs.* TGF-β1 treated group). HaCaT cells were treated with TGF-β1 (5 ng/ml) or with TGF-β1 and SKI2162 (200 nM) for 72 h. The expression level of each gene was analyzed by real-time PCR.

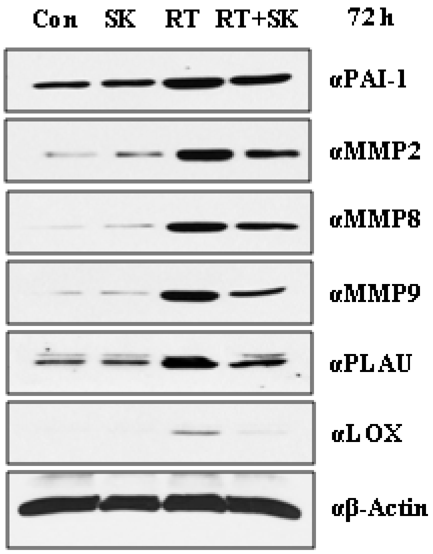

Supplementary Figure 2: The radiation-induced fibrosis markers were suppressed by SKI2162. Protein expression of RIF markers were induced by radiation in the fibroblast cell lines. However, SKI2162 antagonized RIF-related markers. The protein level of each markers was analyzed by western-blot.

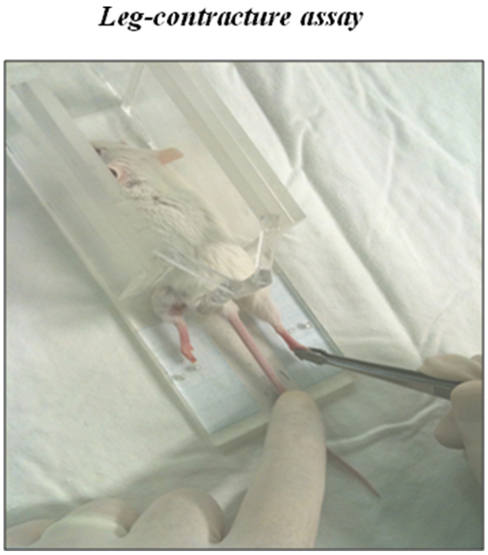

Supplementary Figure 3: The leg-contracture assay. An anesthetized mouse was placed in a Lucite jig, and the length of the extended leg was measured with a ruler inlaid within the base of the jig. The degree of contraction was recorded as the length of the irradiated leg which compared with that of the un-irradiated contralateral leg, expressed as a percentage.

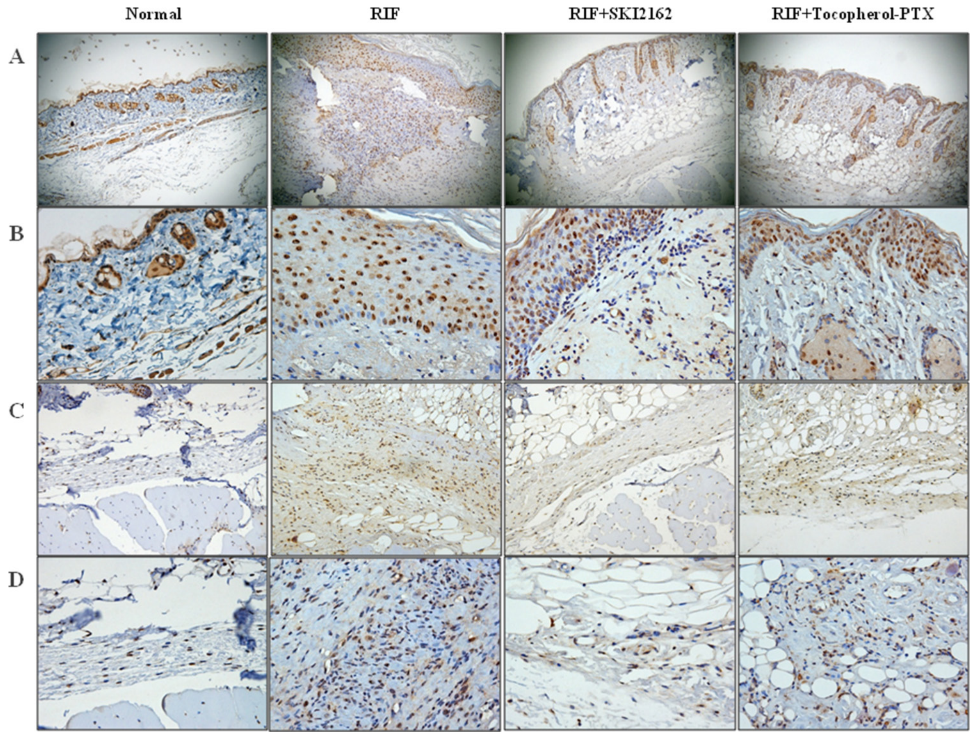

Supplementary Figure 4: Representative IHC images for pSMAD2 in both epidermis (A&B) and dermis (C&D). Images captured under (A) × 100, (B) × 400, (C) × 200, and (D) × 400 magnification respectively. pSMAD2 stained in nucleus both normal and radiation induced fibrotic tissues. However increased expression was detected in vehicle treated RIF tissues especially in subcutaneous layer. In contrast, skin treated with SKI2162 showed less pSMAD2 expression, which confirmed *in vitro* data.

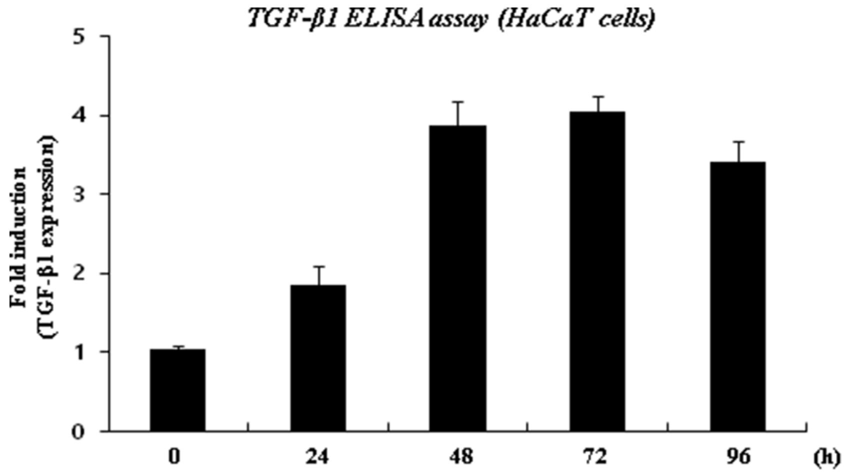

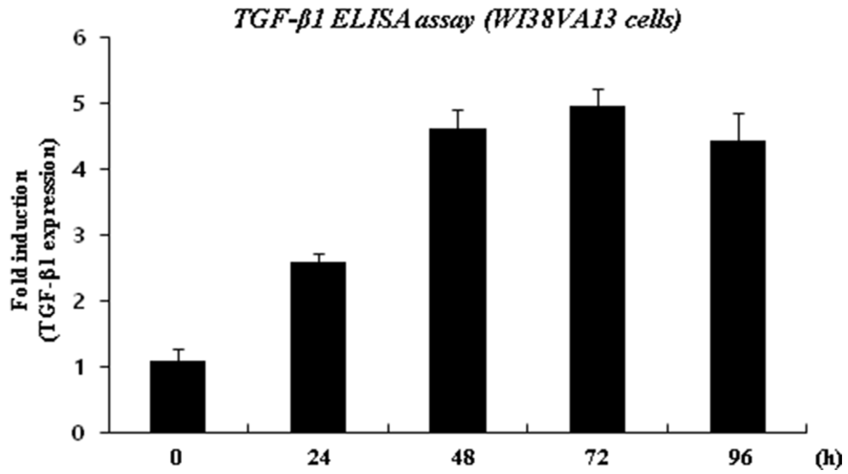

Supplementary Figure 5: TGF-β1 expression was increased by irradiation. After radiation expose, TGF-β1 expression was measured for 96 h by using TGF-β1 ELISA assay kit. TGF-β1 is very highly expressed in WI38 and HaCaT cells at 72 h.

Supplementary Table 1: Selectivity of SKI2162 and LY2157299 against p38 MAPK and ALK1 kinase

	IC_{50}		
	P38 MAPK	ALK1	ALK5
SKI2162	2.206 (21)*	9.082 (73)*	0.094
LY2157299	0.379 (1)*	9.900 (40)*	0.327

*Parenthetic values denote the relative selectivity for the indicated target, calculated by dividing the IC_{50} for the target by the IC_{50} value for ALK5.

Supplementary Table 2: Scoring system for late skin reactions in the radiation induced fibrosis (RIF) mouse model

Score	Description
1.0	No difference from control
1.5	Normal except hair depigmentation
2.0	Hair is depigmented, with sparse regrowth of hair (hair loss 10–30%)
2.5	Sparse regrowth of hair (hair loss 40–70%), no edema, dry skin
3.0	Marked epilation (hair loss 80–100%), thin skin, tight appearance, often has reduction in the size of the limb
3.5	Thin, dry skin, may be edema or dry crust, may be start of skin breakdown
4.0	Focal areas of moist desquamation, many scabs and crusts
4.5	Non-healing ulcers and scabs, some moistness, skin starting to breakdown
5.0	Open wound on limb, draining, full-thickness skin loss
